# A reanalysis: Do hog farms cause disease in North Carolina neighborhoods?

**DOI:** 10.3389/fvets.2022.1052306

**Published:** 2023-02-08

**Authors:** Kaushi S. T. Kanankege, Isaac Traynor, Andres M. Perez

**Affiliations:** ^1^Center for Animal Health and Food Safety, College of Veterinary Medicine, University of Minnesota, St. Paul, MN, United States; ^2^School of Public Health, University of Minnesota, Minneapolis, MN, United States; ^3^College of Veterinary Medicine, Purdue University, West Lafayette, IN, United States

**Keywords:** animal agriculture, public health, CAFO, epidemiology, risk factors, observational studies, correlation

## Abstract

A 2018 publication reported that communities living near hog Concentrated Animal Feeding Operations (CAFO) in North Carolina, USA have increased negative health outcomes and mortalities. While the authors stated that the associations do not imply causation, speculative interpretation of their results by media and subsequent use as evidence in lawsuits caused detrimental effects on the swine industry. We repeated their study using updated data to evaluate the strength of conclusions and appropriateness of methods used with the ultimate goal of alerting on the impact that study limitations may have when used as evidence. As done in the 2018 study, logistic regression was conducted at the individual level using 2007–2018 data, while presumably correcting for six confounders drawn from zip code or county-level databases. Exposure to CAFOs was defined by categorizing zip codes into three by swine density; where, >1 hogs/km^2^ (G1), > 232 hogs/km^2^ (G2), and no hogs (Control). Association with CAFO exposure resulting in mortality, hospital admissions, and emergency department visits were analyzed related to eight conditions (six from the previous study: anemia, kidney disease, infectious diseases, tuberculosis, low birth weight, and we added HIV and diabetes). Re-evaluation identified shortcomings including ecological fallacy, residual confounding, inconsistency of associations, and overestimation of exposure. HIV and diabetes, which are not causally relatable to CAFOs, were also prominent in these neighborhoods likely reflecting underlying systemic health disparities. Hence, we emphasize the need for improved exposure analysis and the importance of responsible interpretation of ecological studies that affect both public health and agriculture.

## 1. Introduction

United States is the third largest swine-producing country in the world (1). North Carolina alongside the Midwestern states of Iowa and Minnesota is the third major swine-producing state in the U.S. (2). The swine (i.e., hog and pig) industry is an economic powerhouse in North Carolina that supports 44,000 total jobs and provides over USD 10 billion in economic output for the state yearly (3). Concentrated animal feeding operations (CAFOs) (4) are the result of a response to the necessity to reduce land use while maintaining the demand for increased food production.

While securing public health should be a primary focus, observational studies that concern public health, agriculture, and the economy need interpretation with caution. Conducting epidemiological analysis and modeling of hazardous environmental exposures in relation to negative health outcomes is extremely challenging. Studies require that the exposure, disease outcomes, and confounding factors are accurately measured and their effects on the exposure and outcome are modeled correctly (5). A 2018 peer-reviewed publication reported that communities living near hog CAFOs have increased negative health outcomes and mortalities, in the U.S. state of North Carolina (6). Authors analyzed the association of all-cause mortality, hospitalizations, and emergency department visits with the CAFO density in a zip code, by conducting a series of independent logistic regression analyses conducted at individual-level, using data from 2007 to 2013, while presumably correcting for six confounding factors. The 2018 study chose six disease conditions (anemia, kidney disease, infant mortality, low birth weight, septicemia, and tuberculosis) that the authors proposed to have associations with CAFO. While authors stated that associations do not imply causation, media and environmental activist groups interpreted the results out-of-context, adding fuel to ongoing debates on hog farming and its impacts on public and environmental health. Consequently, swine producer associations have reported that the published work was used as evidence to file multiple lawsuits causing detrimental effects and financial losses [https://deq.nc.gov/, (7)].

Our objective in this perspective is to share the findings of the re-analysis of the 2018 study using the same yet updated data while using the same methodology; with the intention to evaluate the strength of conclusions and epidemiological appropriateness of the approach. Then, if needed, to propose solutions to the issues identified, interpret, and convey the findings to the interested parties including swine producers and the North Carolina communities. The null hypothesis was that the associations observed in the previous study were appropriate and remained consistent through time, and thus, repeatable. However, in addition to the six disease conditions, we added diabetes and HIV as “controls”; two diseases without known causal associations with CAFOs. HIV and diabetes were chosen because if we observe compatible associations between the residents in zip codes with CAFOs and ending up with these negative health outcomes, then, it is reasonable to theorize that there are underlying health disparities and social determinants of health in these communities leading to health challenges, beyond the proximity to hog CAFOs. As mentioned, linking environmental exposure to negative health outcomes is challenging and we recognize and respect the importance of our neutrality and transparency in the reanalysis. Studying association between exposure to CAFOs and public or environmental health invariably is a complex subject, with many fronts to consider, and no silver bullets. Our overarching goal was to understand whether the hypothesis tested was influenced by a preconceived notion; and whether the previous analysis was capable to answer the epidemiologically intriguing question of whether these communities would have similar negative health issues if there were no hog CAFOs. Through this reevaluation, we intend to encourage the multisectoral collaborations in North Carolina to focus on strategies that would improve the animal, environmental, and public health collectively; and administering efforts and resources to improve the quality of life of the communities.

## 2. Methods

### 2.1. The study area

North Carolina is a southeastern state of the United States (Latitude: 35^o^N, Longitude: 79^o^W). The state inhabits over 10 million people, with 12.9% of persons in poverty (i.e., 11th lowest household income rank among the 50 U.S. states) (8). Raleigh and Charlotte, located in the central and western parts of the state, are the largest metropolitan areas. Rural parts of North Carolina, especially in the mountains and in the east, are affected by persistent inter-generational poverty (9–11). Swine operations are largely concentrated in the southeastern region of the state where over 60% of the state's 8.9 million hogs (2). The swine feeding operations in North Carolina are required to obtain a permit from the North Carolina Department of Environmental Quality confirming the swine waste management system complies with the state requirements [https://deq.nc.gov/; (12, 13)].

### 2.2. Data

Data on negative health outcomes, hog CAFOs, and confounding variables were collected adhering to the data sources described by the previous study (6). The GIS shapefile of North Carolina state and the zip code tabulation areas [*n* = 808 zip codes; (14); https://www.nconemap.gov/]. It is important to note that the previous study used a shapefile with 822 zip codes, whereas, our study used a shapefile with 808 zip codes. This is because zip codes are divisions assigned by the U.S. postal services and are subject to change over time.

Hog CAFO (*n* = 2,248) data including farm location, animal counts allowed, and the number of manure lagoons was extracted from the North Carolina Department of Environmental Quality website [https://deq.nc.gov/; (15)]. The hog operations in North Carolina were defined as “any agricultural feedlot activity involving 250 or more swine with a waste management system, or any agricultural feedlot activity with a liquid animal waste management system that discharge to the surface waters of the state”.

Negative health outcome data on mortality, hospitalizations, and emergency department visits were obtained at a personal/individual level from the Healthcare Cost and Utilization Project's (H-CUP) database (https://www.H-CUP-us.ahrq.gov/). The H-CUP data included State Emergency Department Database (SEDD) (16) and State Inpatient Database (SID) (17) data from 2007 to 2018 (Supplementary Table S1 and Supplementary Figure S1). The SID and SEDD data were checked for any duplicated or erroneous records and merged into one database to be used in the regression analysis. As done in the previous study, the international classification of diseases (ICD) codes were used when choosing the selected disease conditions (Supplementary Table S2).

While no rationale was provided, among the social determinants of health, the previous study included six variables as potential confounders: age, education, median household income, health insurance coverage, number of primary care providers, and smoking prevalence (18). Except for age, which was available from the H-CUP database, the previous study assumed that a person's median household income and other four factors are the same as everyone else in the zip code or county they live. While this flags Ecological Fallacy (19, 20), the same methods were followed. Note that data were gathered at different granularities [(21–25); Supplementary Table S1].

#### 2.2.1. Classifying zip code areas for comparison

**Figure 1:**
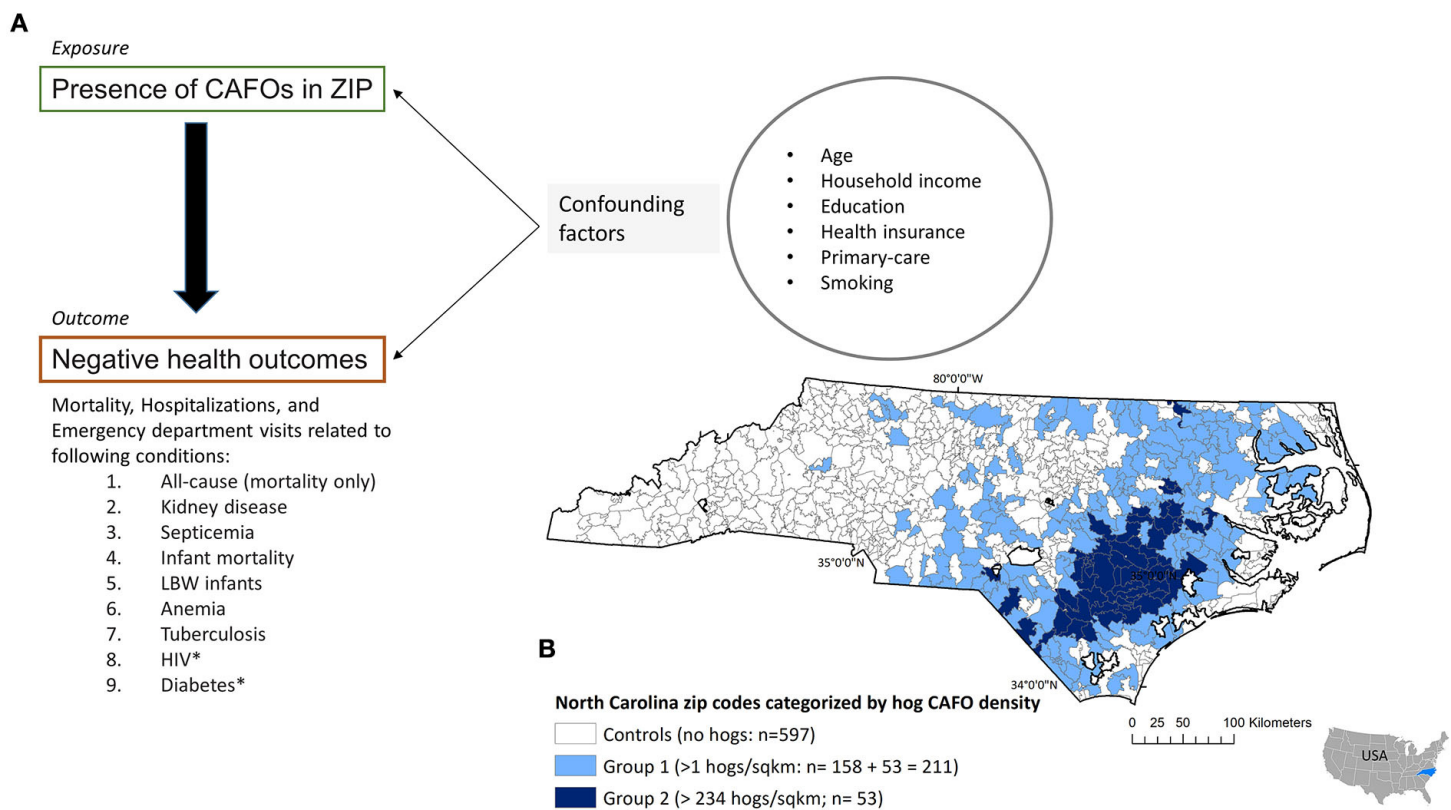
**(A)** Directed acyclic graph denoting the associations between exposure, outcome, and potential confounding factors. **(B)** Division of zip codes in North Carolina based on the swine (i.e., hog) density. The zipcodes were divided into three groups: Group 1 (>1 hogs/square kilometer; *n* = 211), Group 2 (>234 hogs/sqkm; Group 2 is a subset of Group 1), and control zip codes. The threshold of 234 hogs/sqkm was the upper quartile of the hog density [Comparable to the method by Kravchenko et al. (6)].

The hog density per square kilometer was calculated by dividing the number of hogs by the area of the zip code. Comparable to the previous study (6), the zip codes were divided into three study groups based on hog density. The zipcodes with upper quartile of hog density (with >234 hogs per sqkm; *n* = 53) were defined as Group 2; zip codes with ≥ 1 hogs per sq. km were defined as Group 1 (*n* = 211), and the zip codes with zero hog density were considered as the control group (Figure 1). Notice that, Group 2 was included in Group 1. All zip codes without CAFOs were the control group for Group 1. The previous study justified its reason for separating out Group 2 to highlight the magnitude of the effects. The control zip codes for Group 2 were matched at a 1:2 ratio based on propensity score using demographic and socioeconomic characteristics (Supplementary Figure S2). For the step of matching control zip codes for Group 2, a generalized linear model was fitted to obtain the propensity scores, and the greedy matching algorithm was used in the matching process.

Five variables were used to match controls for Group 2 zipcodes: percent of non-Hispanic or Latino black or African-Americans, percent of children (aged 0–19) and people aged 65+ among the residents, median household income, and percent of people with bachelor or higher degree in people aged 25+. All these were continuous variables and they were categorized into five based on natural breaks [Jenks (26)] criteria. Then the zipcodes of Group 2 (*n* = 53) were matched with two control zip codes (*n* = 106). The stratification of the continuous variable is compatible with the stratification of the propensity score approach, which is commonly used in observational epidemiological studies (27–29). However, the reasoning for selecting these factors as matching variables was unclear in the publication by Kravchenko et al. (6). To obtain an unbiased and precise estimation, it is essential to identify matching variables correctly and determine whether the adjustment is needed (30).

The previous study summarized the age-adjusted rates (per 100,000) for all the negative health outcomes comparing the numbers for Group 1, Group 2, and the controls, here, instead of tabulation of these numbers, we summarized the disease data by year for the whole state (Supplementary Figure S1).

#### 2.2.2. Estimation of the risk of exposure based on distance

A simple and concise Directed Acyclic Graph (DAG) is used to clarify the exposure, outcome, and confounding variables (Figure 1). The exposure was the presence of CAFOs in the resident's zip code. Under the outcome of negative health outcomes mortality (both SID and SEDD data), hospitalization (SEDD data), and emergency department visits (SID data) related to all causes, and the eight conditions. In the ecological study design followed by the previous study, the exposure to CAFOs was measured using two methods: 1) the presence of CAFOs in the resident's zip code, and 2) assigning the exposure risk as a function of distance from the CAFOs while accounting for hog number and the human population. The previous study named this distance-based risk calculation “Distance from the Source of potential Contamination (DiSC)” analysis (6). In DiSC analysis, researchers hypothesized that the risk of negative health outcomes is proportional to the number of hogs and calculated the risk of exposure using the number of hogs allowed per farm and the distance from the farm to the center of the census block codes within each zip code. The calculation also assumed all people live at the center of the census block and normalized the calculation using the human population counts. The inclusion of the human population in the calculation makes an artificial weight in the highly populated zip codes and overestimates the exposure. Instead, exposure to an environmental contaminant is commonly quantified using distance-decay functions and there are well-established geostatistical techniques to perform such an analysis: 1) point density estimation weighted by the number of hogs in each farm and 2) geostatistical interpolation technique called co-Kriging (Supplementary Figures S4, S5). However, considering the differences between DiSC analysis and Kriging, in our analysis, we focused only on the exposure assigned based on the presence of CAFOs in the individual's residential zip code, i.e., the main predictor variable of the regression models.

### 2.3. Individual-level logistic regression

A series of individual-level multiple logistic regressions were conducted. As seen in regression equation 1, the main predictor variable was the presence of CAFOs in the zip code, defined as Group 1, Group 2, or controls (X1). The control zip codes were used as the reference to calculate odds ratios (OR) (Figure 1). The outcome encompassed several negative health outcomes including hospitalizations, emergency department visits, and mortalities related to all causes, infant mortality, anemia, kidney diseases, septicemia, tuberculosis, low birthweight of infants, HIV, and diabetes. Analyses were performed for the underlying cause of death (i.e., primary diagnosis) and underlying-plus-secondary causes of death (primary-plus-secondary) diagnoses listed in the H-CUP database. A case-control matched conditional logistic regression was used to compare Group 2 zip codes with the selected and matched control zip codes. The Proc logistic procedure statement of SAS 9.4 software [SAS Institute, Carry NC (31)] was used to conduct the regression analysis and estimate OR, 95% confidence intervals (CI), and frequentist statistical *p*-values (Supplementary material: SAS codes). The ORs relevant to CAFO exposure (i.e., β1) are presented in the results (Table 1 and Supplementary Table S4). The previous publication did not explicitly discuss their approach to addressing low case numbers. In the re-analysis, when encountered with low case numbers (*n* < 30), specifically for tuberculosis, the Proclogit models were evaluated using Hosmer–Lemmeshow goodness-of-fit test. One of the goals of the re-analysis was to add data from additional years and determine if the associations and trends observed by the previous study (data from 2007 to 2013) would hold over time. Therefore, in the reanalysis, H-CUP data from 2007 to 2018 was split into two periods: 1) 2007–2014 (Table 1) and 2) 2015–2018 (Supplementary Table S4). This split year was chosen for ease of comparison with the previous study and H-CUP's adoption of updated ICD codes that occurred in 2015 (ICD-9 to ICD-10).


$$\begin{array}{l} Y=\beta 0+\;\beta 1X1+\;\beta 2X2\;+\;\beta 3X3++\;\beta 4X4\\ \;+\;\beta 5X5\;+\;\beta 6X6+\;\beta 7X7+e\;---(Equation\;1) \end{array}$$

Where,

**Table 1:** Results of the logistic regression models.

**2007–2014**		**Primary diagnosis**		**Primary/Secondary diagnosis**	
**Outcome**	**Disease**	**Study group 1**	**Study group 2**	**Study group 1**	**Study group 2**
**Mortality**	**Anemia**	1.061	1.335^#^	1.130	1.197
		(0.878-1.282; p = 0.543)	(0.802-2.222; p = 0.27)	(0.997-1.281; p = 0.056)	(0.857-1.672; p = 0.29)
	**Kidney disease**	1.100^#^	1.014	1.053	1.136
		(1.029-1.176; p = 0.005)	(0.843-1.221; p = 0.001)	(1.021-1.085; p = 0.001)	(1.047-1.233; p = 0.002)
	**Tuberculosis**	1.213	0.4333	0.977	0.870	
		(0.518-2.840; p = 0.656)	(0.276-68.024; p = 0.29)	(0.476-2.005; p = 0.95)	(0.138-5.469; p = 0.88)
	**Septicemia**	1.105^#^	1.083^#^	1.084^#^	1.073
		(1.076-1.135; p <0.0001)	(1.005-1.167; p = 0.036)	(1.058-1.110; p <0.0001)	(1.004-1.148; p = 0.038)
	**HIV**	1.155	0.668	1.199	0.797
		(0.964–1.383; p = 0.119)	(0.394–1.134; p = 0.135)	(1.029–1.397; p = 0.02)	(0.515–1.233; p = 0.309)
	**Diabetes**	1.003	0.993	1.065	0.863
		(0.861–1.168; p = 0.97)	(0.691–1.425; p = 0.97)	(0.978–1.160; p = 0.149)	(0.691–1.079; p = 0.19)
**Hospital admissions**	**Anemia**	1.087^#^	0.974^#^	0.995^#^	1.013
		(1.069–1.106; p <0.0001)	(0.929–1.021; p = 0.28	(0.986–1.004; p = 0.267)	(0.988–1.039; p = 0.30)
	**Kidney disease**	1.053^#^	1.117^#^	1.109^#^	1.218^#^
		(1.039–1.067; p <0.0001)	1.078–1.158; p <0.0001)	(1.102–1.116; p <0.0001)	(1.198–1.240; p <0.0001)
	**Tuberculosis**	1.555^#^	2.469	1.597	2.431^#^
		(1.348–1.793; p <0.0001)	(1.73303.518; p <0.0001)	(1.415–1.802; p <0.0001)	(1.789–3.303; p <0.0001)
	**Septicemia**	0.962^#^	0.979^#^	0.992^#^	1.007^#^
		(0.953–0.972; p <0.0001)	(0.953–1.006; p = 0.123)	(0.983–1.000; p = 0.0614)	(0.983–1.032; 0.57)
	**LBW**			0.933	0.385
				(0.682–1.276; p=0.663)	(0.168–0.880; p=0.024)
	**HIV**	1.246	1.733	1.195	1.458
		(1.191–1.304; p <0.0001)	(1.531–1.962; p <0.0001)	(1.159–1.231; p <0.0001)	(1.340–1.587; p <0.0001)
	**Diabetes**	1.071	1.133	1.015	1.029
		(1.057–1.084; p <0.0001)	(1.096–1.172; p <0.0001)	(1.006–1.024; p = 0.0014)	(1.005–1.054; p = 0.919)
**Emergency** **dept. visits**	**Anemia**	1.079^#^	1.068^#^	1.124^#^	1.116^#^
		(1.059–1.100; p <0.0001)	(1.014–1.124; p = 0.012)	(1.111–1.138; p <0.0001)	(1.080–1.152; p <0.0001)
	**Kidney Disease**	1.032^#^	0.919	1.25^#^	1.140
		(0.998–1.068; p = 0.067)	(0.840–1.007; p = 0.07)	(1.231–1.269; p <0.0001)	(1.093–1.188; p <0.0001)
	**Tuberculosis**	1.409^#^	1.867	1.684	3.103
		(0.918–2.163; p = 0.116)	(0.534–6.464; p = 0.33)	(1.270–2.233; p = 0.0003)	(1.382–6.966 p= 0.006)
	**Septicemia**	0.905^#^	0.816^#^	0.959^#^	0.843
		(0.867–0.945; p <0.0001)	(0.725–0.919; p = 0.001)	(0.923–0.995; p = 0.026)	(0.761–0.934; p = 0.001)
	**LBW**			1.955	1.956
				(1.571–2.433; p <0.0001)	(1.110–3.449; p = 0.02)
	**HIV**	1.300	2.402	1.235	2.192
		(1.207–1.399; p <0.0001)	(1.992–2.897; p <0.0001)	(1.183–1.290; p <0.0001)	(1.965–2.446; p <0.0001)
	**Diabetes**	1.216	1.521	1.114	1.265
		(1.203–1.229; p <0.0001)	(1.479–1.565; p <0.0001)	(1.107–1.121; p <0.0001)	(1.246–1.285; p <0.0001)

# = Compatible result between previous and current study (<10% difference OR). Odds ratios and 95% confidence intervals of mortality, hospital admissions, and emergency department visits for selected disease conditions from 2007–2014 in North Carolina communities are summarized. Primary and secondary diagnosis listed in the H-CUP database (https://www.H-CUP-us.ahrq.gov/) were analyzed. Study Group 1) represents North Carolina communities in zip codes with >1 hogs/km^2^, Study Group 2) >234 hogs/km^2^, and zip codes without hog CAFOs were the controls (6).

Y = presence or absence of the disease or mortality at individual-level.

β0 = intercept of the logistic regression model fitted.

β1 = the coefficient of the X1 variable. Presented as the odds ratio (Table 1).

X1 = The exposure to CAFOs based on the hog density/sqkm in the zipcode (Categorized as Group 1 where > 1 hog/ sqkm and Group 2 where > 234 hogs/sqkm. Group 1 includes Group 2).

X2 = Age at the individual-level.

X3 = Household income drawn from zipcode-level data.

X4 = Education drawn from zipcode-level data.

X5 = Health insurance drawn from county-level data.

X6 = Primary-care drawn from county-level data.

X7 = Smoking drawn from county-level data.

e = random error term.

#### 2.3.1. Testing for confounding

The logistic regression model with hospitalizations due to kidney disease and septicemia was introduced with the six confounding variables described in the 2018 publication, first one by one and then all six variables together to test for changes in odds ratio with and without them. The resulting OR was compared to the base model. Variables that result in a change of ≥10% of the ORs were considered potential confounders (32). By forcing the six variables that were considered potential confounders into the logistic regression, a conditional logistic regression was fitted to obtain a conditional rather than causal estimation of OR (33).

#### 2.3.2. Sensitivity analysis

A sensitivity analysis was performed by removing the zip codes in cities and urban areas and performing the regression analyses, then the OR was compared to that of the complete dataset. The zip codes in cities (Raleigh and Charlotte) and urbanized areas where the population was ≥ 50,000 were excluded from the individual-level and zip code-level analysis (*n* = 70 zip codes were removed). The previous study reasoned that this exclusion was because CAFOs are predominantly located in rural North Carolina and rural access to medical care is different compared to cities and urban areas.

## 3. Results and discussion

Our re-analysis successfully replicated the individual-level regressions of the previous study [(6), Table 1). The reanalysis unveiled that the study approach was insufficient to assess the intended hypothesis of associations or support the causative associations implied in media reports. The technical soundness of the previous study had several shortcomings including ecological fallacy because the confounding factors were drawn from aggregated data sources, non-adjustment for potential confounders leading to residual confounding, inconsistency of associations when compared between studies and over time, prominence of non-hog CAFO-attributable health conditions, and overestimation of exposure. An ecological study would not confirm or deny exposure over time nor indicate causation. Therefore, instead of ecological studies or survey-based opinion pieces that can be detrimental to the economy (34), we emphasize the need for conducting better epidemiological studies that are spatiotemporally explicit and involve systematic reviews to evaluate the cause-effect over time.

While it is understandable that collecting individual-level data for all the confounding factors in a retrospective study is nearly impossible, this does not justify incorrectly adjusting for the key social determinants of health. Except for age, all other confounding factors were deducted from aggregated data sources (Supplementary Table S1), which indicates a classic case of ecological fallacy. When tested whether these are true confounding variables, the odds ratios in logistic regression models for kidney disease or septicemia hospitalizations did not change more than 10% compared to the simple model (Supplementary Table S5), which indicates none of the six factors are true confounders. Therefore, none of the associations were correctly adjusted for confounding; hence residual confounding.

The variability of associations across diseases and times in terms of the direction of association (ORs >1 or <1) and *p*-value-based statistical significance were inconsistent [comparisons: Table 1 vs. Kravchenko et al. (6) study and Table 1 vs. Supplementary Table S4]. For example, among the 52 associations comparable between studies (excluding HIV and diabetes), the previous study showed 41/52 (79%) statistically significant associations with odd ratios >1, compared to 26/52 (50%) in the re-analysis. The previous study highlighted “In Group 2 zip codes, mortality ORs were 1.50 for anemia (*p* < 0.0001), 1.31 for kidney disease (p < 0.0001), 2.30 for septicemia (*p* < 0.0001), and 2.22 for tuberculosis (*p* = 0.0061) (6).” In the reanalysis, Group 2 mortality OR for primary and secondary diagnoses were 1.197 for anemia (*p* = 0.29; ns i.e., non-significant association), 1.136 for kidney disease (*p* = 0.002), 1.073 for septicemia (*p* = 0.038), and 0.870 for tuberculosis (*p* = 0.88; ns) (Table 1). Tuberculosis OR for Group 2 associations indicated wide confidence intervals with the majority of associations with no statistical significance, which is attributable to the low case numbers. According to the Hosmer-Lemeshow test, all the tuberculosis models for Group 2 were not good model fits and therefore need to be interpreted with caution. Moreover, the association and trends represented by OR and p-values observed in the study period between 2007 – 2014 were not held true and compatible over time, when compared to the 2015 – 2018 study period (Table 1 vs. Supplementary Table S2). From both time periods (i.e., ‘07-‘14 and ‘15-‘18), the general pattern of mortalities in Group 2 residents with primary+secondary diagnosis only resulted in statistically significant OR for septicemia (1.073 and 1.139 in Table 1 and Supplementary Table S4, respectively).

Aside from the unclear rationale or evidence of causation or systematic review behind the choices, the previous study selected five diseases. Especially, human tuberculosis, which is commonly caused by Mycobacterium tuberculosis. Zoonotic tuberculosis (zTB) is commonly related to *M. bovis*; a Mycobacterium species found in cattle, and zoonotic TB of swine origin is extremely rare (35). Tuberculosis is often reported to affect marginalized populations, including racial minorities and migrant or seasonal farmworkers (36, 37). Without the ability to trace back and investigate the potential exposure to tuberculosis, the claim of tuberculosis cases being high in the zip code with CAFOs is not plausible. In our repeated analysis, we included HIV and diabetes, two chronic disease conditions that are not causally relatable to hog CAFOs. Results showed that across the board, HIV and diabetes rates are also higher in these communities (Table 1 and Supplementary Table S4), indicating the need to investigate and account for profound and systemic health disparity in the communities in general.

The key question to answer using an epidemiological approach is the counterfactual scenario of whether these communities would have similar negative health issues if there were no hog CAFOs. A spatial analysis by Son et al. (38), also concluded that the communities near CAFOs are disproportionally affected. However, both studies neglected the alternative explanation that zip codes with hog CAFOs have specific demographic characteristics with greater proportions of African American and American Indian residents, lower median household incomes, fewer residents with a bachelor's degree education level, and fewer primary care providers. These four aspects of race, income, education, and healthcare demographics have all been well established as social determinants of health [Sources: health.gov links, (39–41)]. North Carolina is a state with extreme racial, gender, and income inequities (9, 10, 42). The racially uneven development of the neighborhoods in North Carolina may have disproportionately exposed rural communities to implied agricultural and industrial toxicity (10, 43, 44), which emphasizes the need for focusing solving health disparities in these communities, instead of falsely blaming the agriculture. Previous studies also suggested that while minorities are not directly targeted for exposure to hog farm locations, but are disproportionately exposed, and this disparity may relate to poverty and being a rural population (45).

Through re-analysis, we identified a few ways to improve the assessment. First, in the absence of individual-level confounding variables, the analysis should have been conducted at the zip code or county level, ideally using a spatial regression model. This is because the neighboring administrative units pose an effect on each other and accounting for such spatial dependence is vital when establishing risk factor association (46, 47). Secondly, there are multiple other social determinants of health that need to be considered as confounding factors, and having an index that can represent overall health inequality is the best instead of collecting data from disparate sources (48). The CDC/ATSDR Social Vulnerability Index (49) would be an example of such an indicator where socioeconomic status, household composition, minority status, housing type, and transportation were considered when developing the SVI index. The key differences between our re-analysis and the previous study are that we did not calculate the age-adjusted rate comparison tables, did not conduct *ad-hoc* comparisons of mortalities with other states of the U.S. and did not repeat the DiSC analysis-based logistic regression because we cannot compare that to the geostatistical methods we proposed to better estimate the distance-based exposure. While in our future work we plan to perform the analysis with representative indices of CAFO exposure and include health outcomes and confounding factors obtained from the same granularities, here, we share our perspectives on the need for improved approaches to exposure assessment, analysis, and interpretation of this much-debated study subject.

Hazard identification and risk quantification in environmental epidemiology are challenging (50). Potential environmental contamination including soil, groundwater, and air contamination relatable to CAFOs is a broad topic that cannot be captured using such an ecological study (51, 52). Hog farms are managed by farming systems and farms are different in their management practices, building structure, lagoon structure, and maintenance. Therefore, the use of hog numbers is not an accurate representation of the potential exposure. If the exposure to hog CAFOs is truly enumerated in a spatiotemporally explicit manner, several environmental factors need to be considered and the exposure should be assigned based on farm characteristics, length and routes of potential exposure, and the distance to the residence. Swine waste disposal systems and land application of manure are highly regulated by the North Carolina Department of Environmental Quality and the state legislature [https://deq.nc.gov/; (53–58) and water responsibility (59)]. Hog farming as an industry faces challenges of animal diseases, biosecurity, and securing the health of farm workers while making profits. Changes in farming culture are cost-, labor-, time-, and technology-intensive (60). Regardless, some farming communities are working toward sustainable health and environmental goals (61). Proposing a true One Health approach where human, animal, and environmental health would be a solution for the issue where stakeholders representing the relevant government, swine producers, and community members discuss and plan together multiple solutions and plans for follow-up and follow-through. Hence, we emphasize the importance of responsible interpretation of ecological studies that concerns public health, agriculture, and economy to support the ultimate goal of directing the state's efforts and resources toward factors and determinants truly associated with diseases.

## Data availability statement

The datasets presented in this article are not readily available because the health data are available for purchase via the Healthcare Cost and Utilization Project's (H-CUP) database (https://www.H-CUP-us.ahrq.gov/). Requests to access the health datasets should be directed to (hcup@ahrq.gov). The hog CAFO data are open and accessible via North Carolina Department of Environmental Quality web site (https://deq.nc.gov/). The data on confounding factors are all available open access from relevant data sources listed in Supplementary Table S1. The exact open-source data that were used in the re-evaluation study can be provided on request (kanan009@umn.edu).

## Ethics statement

The Institutional Review Board of the University of Minnesota determined that the research activity of re-evaluation of Kravchenko et al. (6) does not involve human subjects as defined by DHHS and FDA regulations (IRB ID: STUDY00010813). De-identified health data were obtained from the H-CUP database and were stored and analyzed in accordance with the data security measures determined by the University of Minnesota.

## Author contributions

KK contributed to the research design, supervised the data cleaning, conducted the analysis, and compiled the manuscript. IT conducted the data cleaning and analysis on SAS and edited the manuscript. AP proposed the research design and contributed to the interpretation and discussion of the results. All authors contributed to the article and approved the submitted version.
