# Supplementary material for: A reanalysis: Do hog farms cause disease in North Carolina neighborhoods?
Source: Front Vet Sci. 2023 Feb 8;9:1052306. doi: 10.3389/fvets.2022.1052306 (PMC9945130; doi:10.3389/fvets.2022.1052306)
Supplement: Supplementary file 1 [file Data_Sheet_1.docx]

# **Supplementary material**


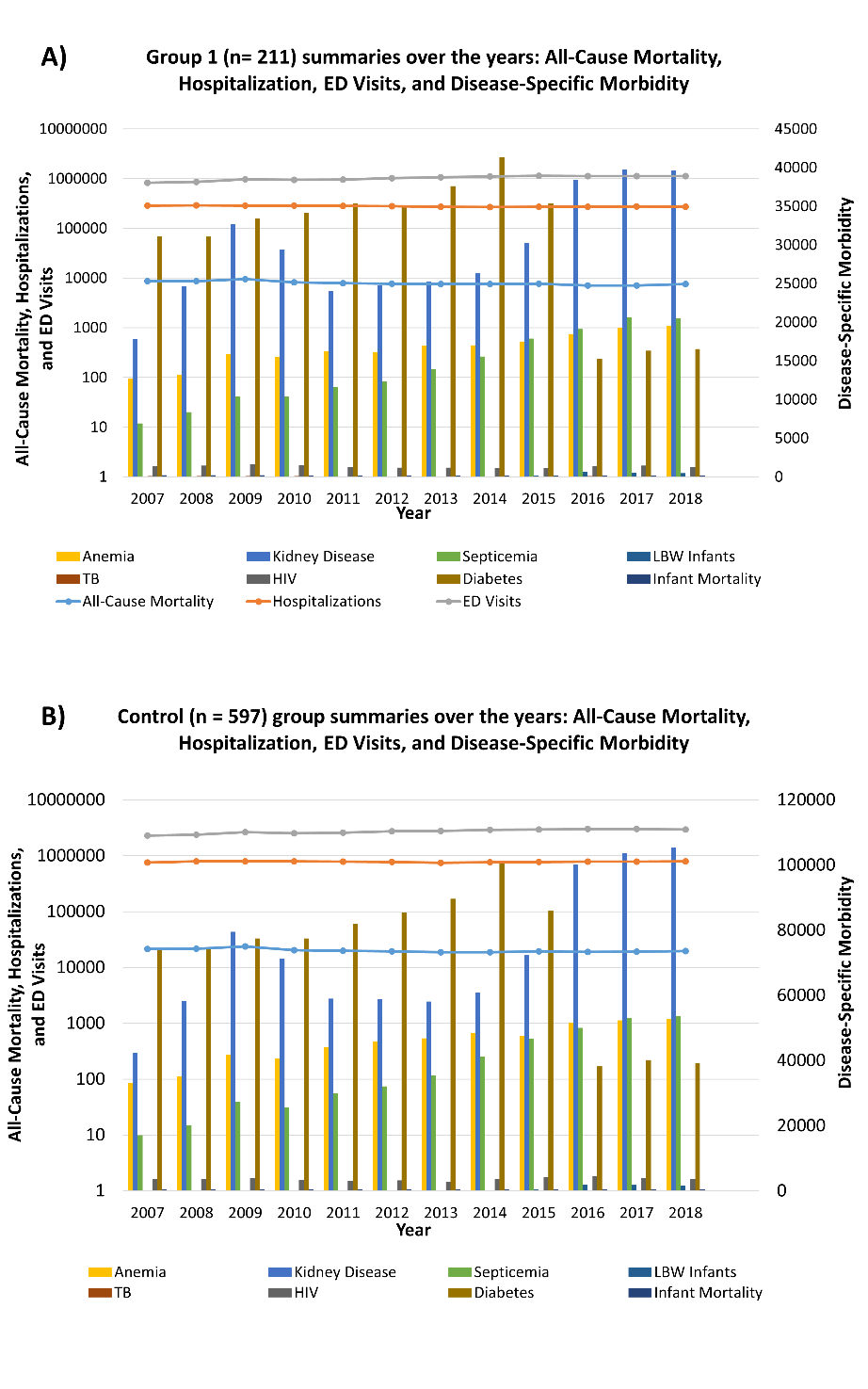


**Figure S1**. Comparison of the case numbers between A) study group 1 (i.e. 211 zip codes with >1 hogs/sqkm) and B) controls (i.e. 597 zip codes with no hogs) over time. The negative health outcomes in North Carolina including all-cause mortality, hospitalizations, emergency department visits, and disease-specific mortalities related to eight selected disease conditions over the span of 2007 – 2018 obtained from: SEDD and SID of Healthcare Cost and Utilization Project’s (H-CUP) database are summerized (<https://www.H-CUP-us.ahrq.gov/>).

**Interpretation:** When the primary diagnosis listed, in H-CUP data were summarized by year, the all-cause mortality, hospital admissions, and emergency department visits did not exhibit a trend over the years. The disease specific morbidities (both hospital and emergency department visits collectively) related to anemia and septicemia exhibited an increase in the rates in both Group 1 and Control zip codes. Kidney diseases rates were less in the years 2011 – 2014 compared to the other years.

**
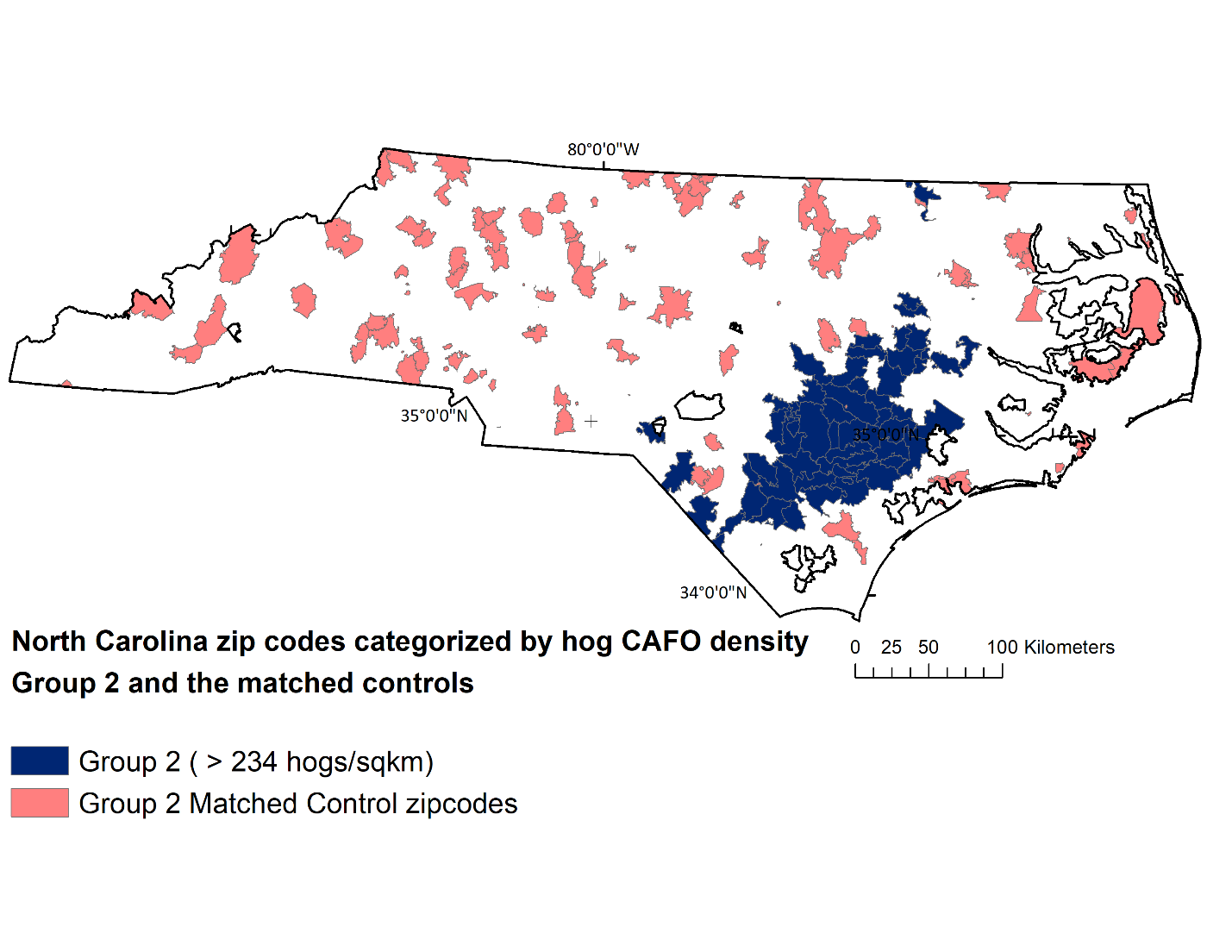
**

**Figure S2**. Study Group 2 zip codes and the matched controls for the Group 2 zip codes.

**Table S1**. Summary of variables and data sources.

| Variable | Time | Granularity | Source | Attributes/ Comments |
| --- | --- | --- | --- | --- |
| CAFOs | 2020 | Farm-level | NC Dept. of Environmental quality (DEQ) | Farm location, number of animals, number of manure lagoons |
| Negative health outcomes | 2007 - 2018 | Individual-level | The Healthcare Cost and Utilization Project (HCUP)   - Emergency dept: SEDD - SID: Hospitalizations | Age, gender, date, diagnosis, resolution |
| Confounding factors |  |  |  |  |
| - Age | 2007 – 2018 | Individual-level | HCUP |  |
| - Household income* | 2011 - 2016 | Zipcode-level | American Community Survey |  |
| - Education* | 2011 - 2016 | Zipcode-level | American Community Survey |  |
| - Health insurance^#^ | 2010, 2015 - 2018 | County-level | Area Health Res. Files (AHRF) |  |
| - Primary-care^#^ | 2010 - 2018 | County-level | Area Health Res. Files (AHRF) |  |
| - Smoking prevalence^¥^ | 2005 – 2012, 2017 | County-level | Behavioral Risk Factor Surveillance System (BRFSS); County Health Rankings | Model-based estimates of adult smoking prevalence (https://www.countyhealthrankings.org/ |

*Zip code-level data on median household income and education, defined as the percentage of people aged 25+ who attained an education level higher than a bachelor’s degree were obtained from the 2011 – 2016 American community survey (ACS, 2020).

# County-level data on percentage of those who are under 65 years of age without health insurance (F1475110 - F1547418) and number of primary care providers, specifically physicians (F1467510 - F1467518), were obtained from the Area Health Resources Files (AHRF) for the years 2010 – 2018, values were averaged across the years (AHRF, 2021). County-level data on percentage of those who are under 65 years of age without health insurance (F1475110 - F1547418) and number of primary care providers, specifically physicians (F1467510 - F1467518), were obtained from the Area Health Resources Files (AHRF) for the years 2010 – 2018, values were averaged across the years (AHRF, 2021). Given the definition for ‘primary care providers’ used in the previous study (Kravchenko et al., 2018) was not mentioned, in this re-analysis, only physicians were used as per the definition provided by Healthcare.gov (Specific variable name: Phys, Primary Care, Patient Care; Healthcare.gov).

^¥^While no further details were described, (Kravchenko et al., 2018) claims to have obtain current smoking prevalence at county-level using Behavioral Risk Factor Surveillance System (BRFSS) data (BRFSS CDC, 2020). BRFSS surveys are a random digit–dial telephone (cellular and landline) survey of noninstitutionalized adults aged 18 years or older, conducted through collaborations between the Centers for Disease Control and Prevention (CDC) and all 50 US states. As per the published literature, unless extraneous steps such as those by Song et al., (2016), which uses a Bayesian geostatistical approach to estimate the smoking prevalence using BRFSS survey data, it is not straightforward to calculate smoking prevalence using BRFSS data. Thus, in this re-analysis study, we used the model-based county-level estimates of ‘percentage of adults who are current smokers (age adjusted)’ available from County Health Rankings database (www.countyhealthrankings.org; PLACES project; Adult smoking). Specifically, the county-level smoking prevalence estimates for 2014 (which covered the BRFSS survey data from 2006 – 2012) and 2017 were downloaded.

**Table S2**. The International Classification of Diseases (ICD) codes used to extract relevant disease diagnostics from the H-CUP database. See Appendix 1 of Kravchenko et al., (2018) for the compatible table.

|  | **Disease Condition** | **ICD-9 Codes** | **ICD-10 Codes** |
| --- | --- | --- | --- |
| 1 | Anemia | 280-285 | D50-D53, D55-D59, D60-D64 |
| 2 | Kidney Disease | 580-589 | N00-N19 |
| 3 | Tuberculosis | 010-018 | A15-A19 |
| 4 | Septicemia | 038, 995.91 | A40-A41 |
| 5 | Low Birth Weight | V21.3 | P07.1 |
| 6 | HIV | 042 | B20 |
| 7 | Diabetes | 250 | E10-E11, E13 |

**Table S3**. The summaries of five matching variables used to match the study Group 2 with the control zip codes. See Table S1 of Kravchenko et al., (2018) for the compatible table.

|  | **Variable** | **Group 2 zipcodes** | **Matched Control zipcodes for Group 2** |
| --- | --- | --- | --- |
| 1 | Percent of African Americans | 29.23 ± 15.47 | 29.33 ± 16.05 |
| 2 | Percent of children (aged 0-10) | 19.10 ± 1.02 | 18.25 ± 2.83 |
| 3 | Percent of people aged 65+ | 11.05 ± 2.35 | 12.94 ± 10.04 |
| 4 | Median household income | $37,111.17 ± 7304.96 | $40,540. 63 ± 11, 686. 90 |
| 5 | Percent of people with high school or higher education in people aged 25+ | 18.57 ± 3.18 | 17.54 ± 5.01 |

**Table S4**. Results of the logistic regression models. Odds ratios and 95% confidence intervals of mortality, hospital admissions, and emergency department visits for selected disease conditions from 2015 - 2018 in North Carolina communities are summarized. Primary and secondary diagnosis listed in the H-CUP database ([https://www.H-CUP-us.ahrq.gov/](https://www.hcup-us.ahrq.gov/)) were analyzed. Study Group 1) represents North Carolina communities in zip codes with >1 hogs/km^2^, Study Group 2) > 234 hogs/km^2^, and zip codes without hog CAFOs were the controls (reference).

#=Compatible result (<10% change) with 2007-2014 results (Table 2)

**Table S5.** Testing for confounding. Results of logistic regression indicating odds ratios and the percent change of odds ratios when each of the six confounding variable was introduced to the model one-by-one and then collectively. The table summarizes the association of exposure Group 1 (compared to the controls) for hospitalizations due to kidney diseases and septicemia.

Example interpretation: The OR of kidney disease in Group 1 compared to the control zip codes, without adjusting for confounding factors is 1.144. When the confounding factor ‘Education’ was added to the logistic regression, the new OR for kidney disease is 1.051. The percent change of OR was (1.144 – 1.051)/1.144 * 100 = 8.13%. This is <10% change in OR, which is commonly considered an indicator that the newly added variable is a confounding factor that influences both independent variable (exposure to CAFOs) and the dependent variable (disease outcome).

**Table S6.** Sensitivity analysis with and without the urban zipcodes. Odds ratios (OR) of mortalities related to the eight conditions are compared. There were no changes in the OR.

|  | **Negative health outcome** | **With urban areas** | **Without urban areas** |
| --- | --- | --- | --- |
| **Mortality** | **Anemia** | 1.165 | 1.165 |
|  | 95% CI & P | (0.748-1.813; p=0.499) | (0.748-1.813; p=0.499) |
|  | **Kidney Dz** | 1.179 | 1.179 |
|  | 95% CI & P | (1.057-1.315; p=0.003) | (1.057-1.315; p=0.003) |
|  | **Tuberculosis** | 0.741 | 0.741 |
|  | 95% CI & P | (0.193-2.847; p=0.66) | (0.193-2.847; p=0.66) |
|  | **Septicemia** | 1.070 | 1.070 |
|  | 95% CI & P | (1.036-1.106; p<0.0001) | (1.036-1.106; p<0.0001) |
|  | **HIV** | 1.092 | 1.092 |
|  | 95% CI & P | (0.723-1.647; p=0.676) | (0.723-1.647; p=0.676) |
|  | **Diabetes** | 0.913 | 0.913 |
|  | 95% CI & P | (0.726-1.148; p=0.438) | (0.726-1.148; p=0.438) |
|  | **All-cause** | 0.913 | 0.913 |
|  | 95% CI & P | (0.726-1.148; p=0.438) | (0.726-1.148; p=0.438) |


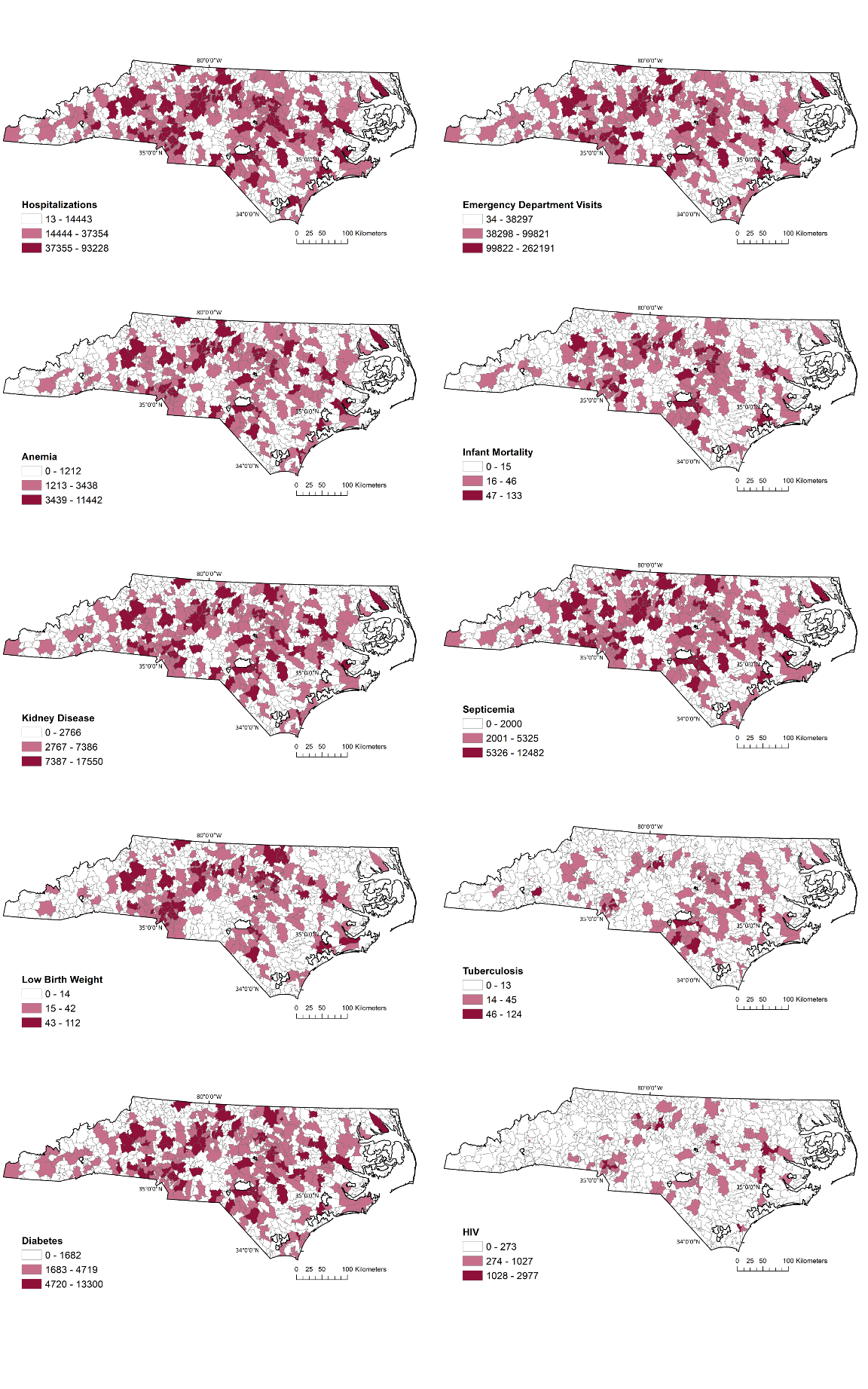


**Figure S3.** Mapped summaries of the hospitalizations, emergency department visits, and the rates of selected disease conditions. H-CUP data from 2007 – 2014 were used in the summaries. Each variable is categorized in to three based on the natural breaks (Jenks) if the variable.

### **Estimation of the risk of exposure based on distance**

In the ecological study design followed by previous study, the exposure to CAFOs was measured using two methods: 1) presence of CAFOs in the resident’s zip code, and 2) assigning the exposure risk as a function of distance from the CAFOs while accounting for hog number and the human population. Previous study named this distance based risk calculation ‘Distance from the Source of potential Contamination (DiSC)’ analysis (Kravchenko et al., 2018). In DiSC analysis, researchers hypothesized that the risk of negative health outcome is proportional to the number of hogs and calculated the risk of exposure using the number of hogs allowed per farm and the distance from the farm to the center of the census block codes with in each zip code. The calculation also assumed all people live at the center of the census block and normalized the calculation using the human population counts. Inclusion of human population in the calculation makes an artificial weight in the highly populated zipcodes and overestimate the exposure. Instead, exposure to an environmental contaminant is commonly quantified using distance-decay functions and there are well-established geostatistical techniques to perform such analysis: 1) point density estimation weighted by the number of hogs in each farm and 2) geostatistical interpolation technique called co-Kriging. Point density analysis generates raster cells in which the neighborhood values are calculated using the points (farms in this case) falls within the defined cell size. Kriging can be understood as a two-step process, where, step 1 is fitting the spatial variogram or likelihood for the data observed at the sampled points; and step 2 involves the interpolation of values for unsampled points using the weights derived from this covariance structure (Isaaks and Srivastava, 1989; Kanankege et al., 2020). In co-Kriging, we used both number of hogs and the number of lagoons to calculate the distance based exposure. In the context of this study, the co-Kriging estimates can be defined as a spatially continuous variable that represent the risk of exposure to CAFOs as a function of distance from all the CAFOs, in which the hog numbers and number of lagooons at close proximity would contribute to the exposure estimate more than those that are distant.

**
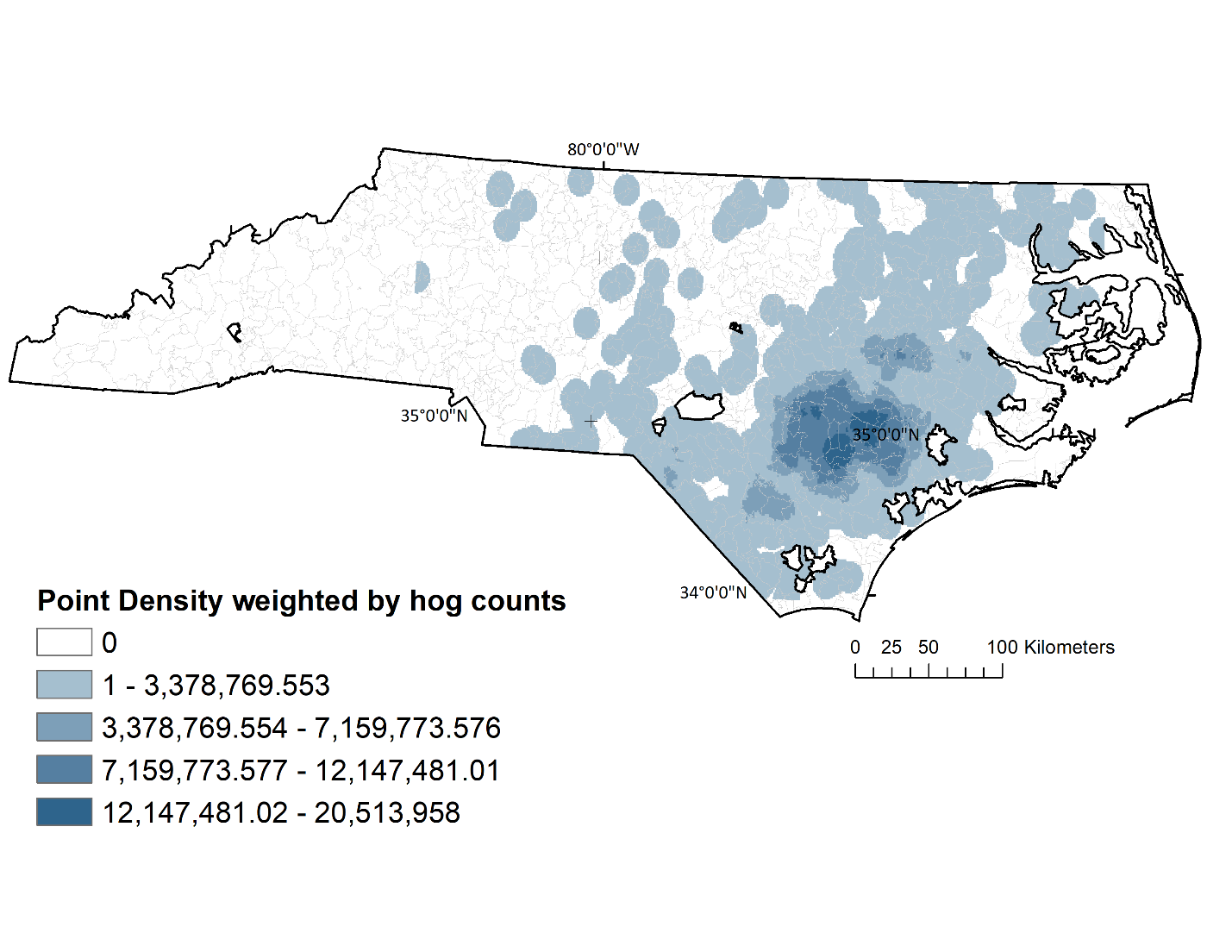
**

**Figure S4.** Point density map of hog CAFOs in North Carolina weighted by hog counts. The zip code map is overlaid on the point density map and the areas with zero density is separated as an independent category.


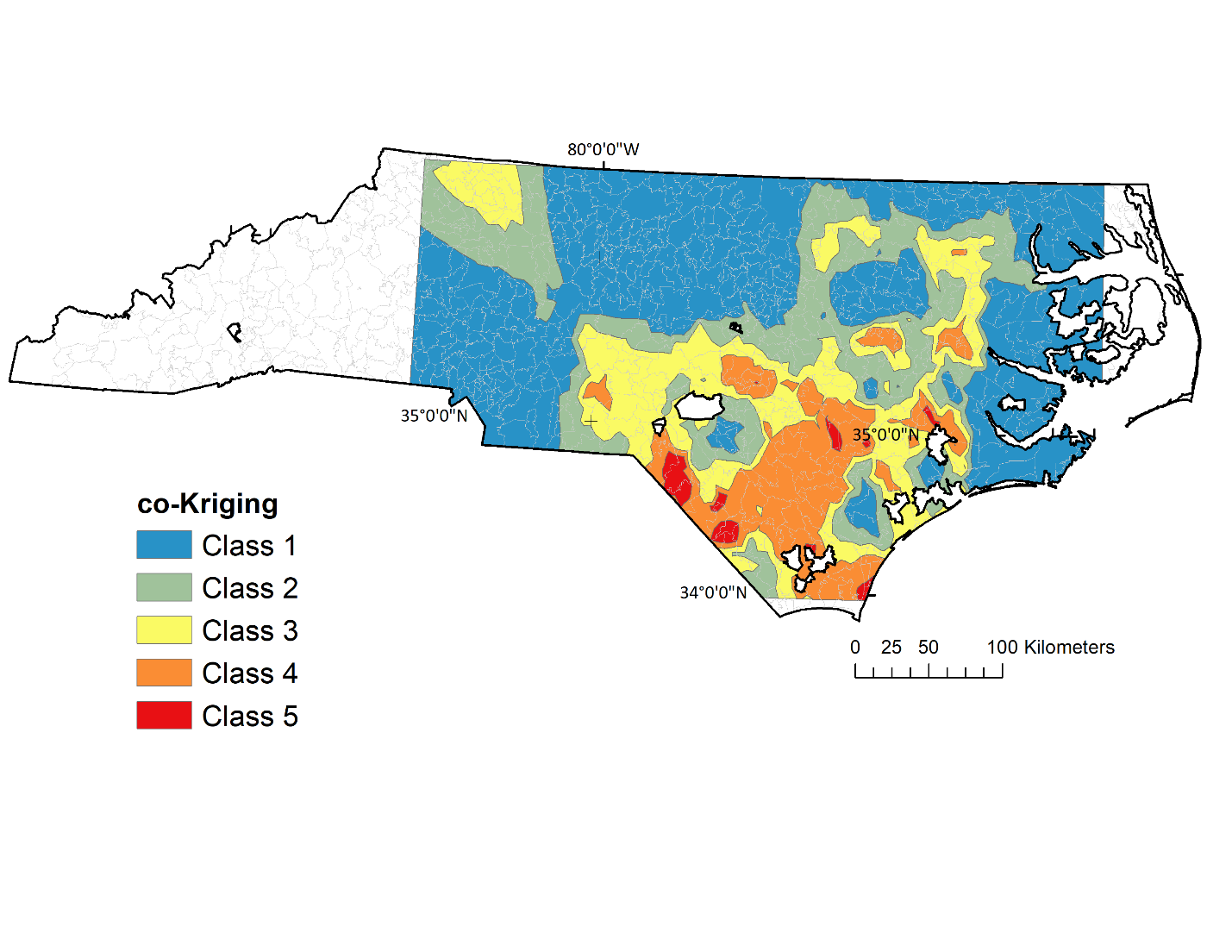


**Figure S5**. Map of Co-Kriging using two variables: Var 1) the 10-log of hog counts and Var 2) number of lagoons. The predicted exposure levels are classified into five based on quantiles in which class 5 represents the highest exposure and class 1 the lowest. Co-Kriging is a geostatistical method and the distance between CAFOs and the spatial dependence is accounted for in the calculation. Further details on co-Kriging method are found elsewhere (Isaaks and Srivastava, 1989; Kanankege et al., 2020).

**Supplementary documents: SAS codes**

/*SAS Code used for logistic regression and odds ratio determination; Code below uses the Kidney Disease variable as an example*/

**data** hcup;

set hcup.hcup0714;

**run**;

/*Group 1 DX1 Comparison Code*/

ods select parameterestimates oddsratios; /*Select output*/

**proc** **logistic** data=hcup; /*Logistic regression procedure*/

class studyG1 (desc); /*studyG1 is a character variable that indicates zip codes as either 1 (Study Group 1) or 0 (Control)*/

model died (event='1') = studyG1 AHRF_Primary_Care AHRF_Health_Insurance BRFSS_Smoking2012 Age MedianHHIncome Education; /*Defines the logistic regression model and includes confounding variables*/

where DX1_Kidney = **1**; /*Only analyzes cases of primary diagnosis of kidney disease*/

title 'Death: DX1 Kidney - Group 1';

**run**;

ods select parameterestimates oddsratios;

**proc** **logistic** data=hcup;

class studyG1 (desc);

model DX1_Kidney (event='1') = studyG1 AHRF_Primary_Care AHRF_Health_Insurance BRFSS_Smoking2017 Age MedianHHIncome Education;

where Hospital = **1**;

title 'Hospitalizations: DX1 Kidney - Group 1';

**run**;

ods select parameterestimates oddsratios;

**proc** **logistic** data=hcup;

class studyG1 (desc);

model DX1_Kidney (event='1') = studyG1 AHRF_Primary_Care AHRF_Health_Insurance BRFSS_Smoking2017 Age MedianHHIncome Education;

where EDV = **1**;

title 'Emergency Dept: DX1 Kidney - Group 1';

**run**;

/*Group 1 DX1DX2 Comparison Code*/

ods select parameterestimates oddsratios;

**proc** **logistic** data=hcup;

class studyG1 (desc);

model died (event='1') = studyG1 AHRF_Primary_Care AHRF_Health_Insurance BRFSS_Smoking2017 Age MedianHHIncome Education;

where DX1DX2_Kidney = **1**;

title 'Death: DX1DX2 Kidney - Group 1';

**run**;

ods select parameterestimates oddsratios;

**proc** **logistic** data=hcup;

class studyG1 (desc);

model DX1DX2_Kidney (event='1') = studyG1 AHRF_Primary_Care AHRF_Health_Insurance BRFSS_Smoking2017 Age MedianHHIncome Education;

where Hospital = **1**;

title 'Hospitalizations: DX1DX2 Kidney - Group 1';

**run**;

ods select parameterestimates oddsratios;

**proc** **logistic** data=hcup;

class studyG1 (desc);

model DX1DX2_Kidney (event='1') = studyG1 AHRF_Primary_Care AHRF_Health_Insurance BRFSS_Smoking2017 Age MedianHHIncome Education;

where EDV = **1**;

title 'Emergency Dept: DX1DX2 Kidney - Group 1';

**run**;
